# Effects of Annual Growth Conditions on Phenolic Compounds and Antioxidant Activity in the Roots of *Eryngium montanum*

**DOI:** 10.3390/plants12183192

**Published:** 2023-09-06

**Authors:** Mónica L. Pérez-Ochoa, Araceli M. Vera-Guzmán, Demetria M. Mondragón-Chaparro, Sadoth Sandoval-Torres, José C. Carrillo-Rodríguez, Netzahualcoyotl Mayek-Pérez, José L. Chávez-Servia

**Affiliations:** 1 CIIDIR-Oaxaca, Instituto Politécnico Nacional, Gustavo A. Madero, Ciudad de Mexico 07320, Mexico; mperezo1800@alumno.ipn.mx (M.L.P.-O.); dmondragon@ipn.mx (D.M.M.-C.); ssandovalt@ipn.mx (S.S.-T.); 2 Instituto Tecnológico del Valles de Oaxaca, Santa Cruz Xoxocotlán, Oaxaca 71233, Mexico; jcarrillo_rodriguez@hotmail.com; 3 Unidad Académica Multidisciplinaria Reynosa, Universidad Autónoma de Tamaulipas, Reynosa 88710, Mexico; nmayeklp@yahoo.com.mx

**Keywords:** environmental factors, medicinal plants, secondary metabolites, *Eryngium*

## Abstract

Medicinal plants grown in natural settings are exposed to different adverse environmental conditions that determine their growth and development as well as the composition and concentration of secondary metabolites in their organs. The objective of this study was to evaluate the effects of environmental conditions associated with localities and annual growth cycles on the contents of phenolic compounds and flavonoids, antioxidant activity and potentially bioactive phenolic acids in the roots of *Eryngium montanum*, a medicinal species from temperate Mexico. The samples for composition analysis were collected using a bifactorial design: Factor A consisted of the localities (Morelos and La Unión de San Martin Huamelulpam, Mexico) and Factor B was represented by the annual growth cycle (2020 and 2021). In each sample, the contents of polyphenols and equivalent flavonoids of quercetin and catechin and antioxidant activity were evaluated using spectrophotometry. Subsequently, chlorogenic, caffeic and rosmarinic acids were identified and quantified using high-performance liquid chromatography with diode-array detection (HPLC-DAD). The annual growth conditions and, to a lesser extent, the locality of origin of the samples significantly influenced the contents of phenolic compounds and antioxidant activity. The environmental conditions that occurred in 2021 favored an increase in the contents of phenolic compounds compared to those in 2020, and the same pattern was observed for chlorogenic acid; however, for caffeic and rosmarinic acids, the opposite pattern was observed. The content of phenolic acids in the roots of *E. montanum* follows different and independent patterns between cycles based on the interaction between the locality of origin and annual growth cycle. This study quantifies the magnitude of the total environmental effect on the phenolic compound concentrations in *E. montanum* roots, which was measured via sampling during two annual growth cycles, where the sampling locations factor had little influence. The bioactive compounds identified in *E. montanum* roots have the potential for use as alternative medicines, as mentioned by different families from Oaxaca, Mexico.

## 1. Introduction

The genus *Eryngium* (Apiaceae, subfamily Saniculoidea) includes more than 250 species distributed in temperate and tropical regions of North Africa, Mediterranean Europe, Southwest Asia, Australia and the Americas; approximately two-thirds of all the species have a high incidence rate in Mexico and South America [1]. The aerial parts and roots of different *Eryngium* species are used in traditional medicine for the treatment of diabetes, dyslipidemia, hypertension, malaria, kidney disease and infectious diseases of the gastrointestinal tract in countries such as Turkey, Iran and Mexico, have been shown to have hypoglycemic, analgesic, anti-inflammatory, anticancer, antimicrobial, antihemolytic, renoprotective, antihypoxic, cytotoxic, contractive, phytotoxic and antioxidant properties or activities and are even useful in food preservation [2,3,4]. For example, Cortés-Fernández et al. [5] determined that *Eryngium maritimum* L. rhizome extracts express an anti-inflammatory effect in biological models with Jurkat cells through antioxidant effects and reductions in pro-inflammatory gene expression, and Vaez et al. [6] revealed antidiabetic effects through reductions in sugar levels in rat models with induced diabetes by using hydroalcoholic extracts of *Eryngium thyrsoideum* and *E. caucasicum*.

In in vitro culture tests of *Eryngium viviparum*, Ayuso et al. [7] observed higher contents of phenolic compounds and antioxidant activity levels in extracts from roots (> 60%) than in extracts from aerial parts, and the most abundant compounds were *trans* rosmarinic acid and *trans* 3-O-caffeoylquinic acid. However, these results are not common across *Eryngium* species, as documented by Kikowska et al. [8] for in vitro cultures of *E. maritimum* and *E. planum,* in which the opposite tendencies were observed. Root composition evaluations of *Eryngium* species have indicated that these organs contain triterpene saponins, glycosides of hydroxycinnamic acids (e.g., p-coumaric, ferulic and caffeic acids), flavonoids such as catechin and epicatechin [9] and phenolic acids such as rosmarinic and chlorogenic acids [10] that together confer high antioxidant activity.

*E. montanum* J.M. Coult. & Rose is a common biennial or perennial herb distributed worldwide in subtropical and temperate regions but is considered endemic to central and south–southeastern Mexico, where it is usually associated with grass ecosystems in the disturbed zones of pine–oak and coniferous forests [11,12]. This species is used for the treatment of gastrointestinal, kidney and digestive problems in Mexico and Latin America [13,14], and to date, *E. montanum* has not been included on the Red List of the International Union for Conservation of Nature (IUCN) [15]. Traditional medicine associates the properties and efficacy of plants based on previous knowledge transmitted orally and/or learned by individuals with the treatment of organic or clinical diseases and ethno-diseases [3,16]. The efficacy or curative ability of plants depends on their phytochemical composition, but the composition of secondary metabolites varies as a function of ontogenetic, genetic and micro- and macroenvironmental factors and their interactions on temporal and spatial scales according to inter- and intraspecific diversity, ecophysiology, the ecological–geographic context and functional metabolic processes underlying plant responses [17,18].

Wild medicinal plants synthesize secondary metabolites (e.g., flavonoids and phenols) to counteract the effects of biotic and abiotic factors. These compounds change in affectation grade from one year to the next and influence the biosynthesis, translocation, and accumulation of metabolites in different organs of the plant [19,20,21]. Additionally, it is important to consider the complex and multilevel mechanisms of reactive oxygen species (ROS) as a response of the plant to any stress condition. In this context, this work aimed to evaluate the effect of environmental conditions associated with localities and annual growth conditions on the contents of phenolic compounds and flavonoids and antioxidant activity levels in the roots of *Eryngium montanum*, a medicinal species found in temperate climates of south–southeastern Mexico and used by indigenous communities for the treatment of gastrointestinal disorders (e.g., diarrhea and stomach pains) [14].

## 2. Results

### 2.1. Total Phenolic Compounds and Antioxidant Activity Evaluated Using Spectrophotometry

Analysis of variance revealed significant effects (*p* < 0.01) of annual cycles (C) on the contents of polyphenols and flavonoids and antioxidant activity, of populations or sample origins (Po) on the contents of quercetin-equivalent flavonoids and antioxidant activity evaluated using FRAP and of the cycle–population interaction (C × Po) on the content of polyphenols and antioxidant activity evaluated using FRAP in the methanolic extracts of *E. montanum* roots. It was also observed that for the contents of equivalent flavonoids of quercetin and catechin and antioxidant activity evaluated using DPPH, the effect of the annual cycle was independent of the effect of the location or sampling site (Po) of *E. montanum*. In addition, the magnitudes of the variances due to annual cycles and sample origins had stronger significant effects than the effect of the Po × C interaction (approximation equivalent to the genotype x environment effect) (Table 1).

In this work, an estimate of the environmental or ecogeographic niche effect was obtained by sampling the same sites, populations, and localities in two consecutive years (2020 and 2021), and it was assumed that under natural conditions, the weather varied from one year to another at the sampling sites. The evaluation of phenolic compounds and antioxidant activity showed that the samplings carried out in 2021 yielded a higher concentration of phenolic compounds (from 13.2 to 33.7%) than those in 2020; this pattern was also reflected in the antioxidant activity (8.8 to 18.7%). Between the localities of origin of the samples and populations (Po), there were no significant differences in the contents of total polyphenols or catechin-equivalent flavonoids or antioxidant activity evaluated using DPPH, which indicates that from one population to another or from one site to another, the concentration of these compounds did not change significantly. The only differences observed were in the contents of quercetin-equivalent flavonoids and antioxidant activity evaluated using FRAP in favor of the Morelos locality. The significant interaction between the localities of origin of the samples (Po) and annual cycles (C) for the total contents of polyphenols and antioxidant activity evaluated using FRAP indicated that during 2021, the annual climatological, edaphic and microenvironmental conditions significantly influenced the concentration of phenolic compounds in the sampled roots of *E. montanum* (Table 2).

Pearson correlation analysis of the contents of polyphenols and flavonoids and the antioxidant activity evaluated using the DPPH and FRAP methods per year of sample collection revealed significant positive correlations for the samples collected in 2020 (0.70 < r < 0.93; *p* < 0.001) and 2021 (0.74 < r < 0.94; *p* < 0.001). This indicates that regardless of the annual variations in growth conditions during the sampling years, the phenolic compounds present in the roots of *E. montanum* contributed to the estimated antioxidant capacity in each sample and were associated with the medicinal effect that the communities attribute to these plants.

### 2.2. Phenolic Acids Evaluated Using HPLC-DAD

**Figure 1:**
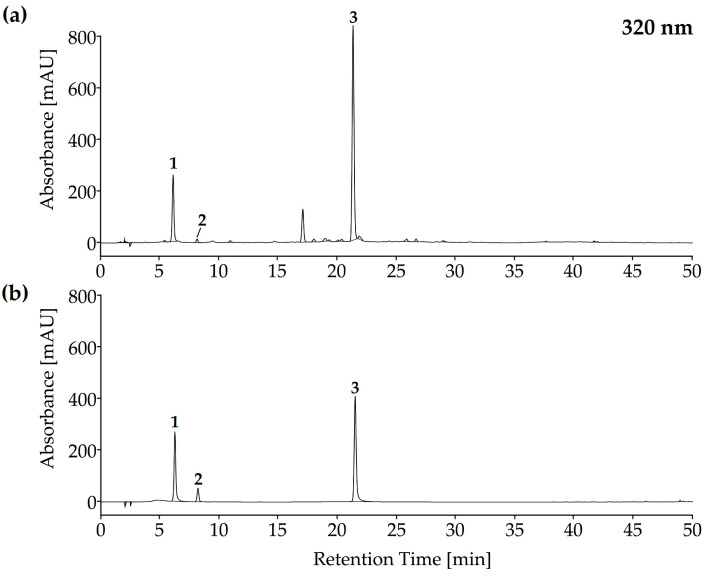
Chromatographic profile based on HPLC-DAD analysis of phenolic acids in methanolic extracts from roots of *E. montanum* (**a**) and based on the reference standards (**b**): (1) chlorogenic acid, (2) caffeic acid and (3) rosmarinic acid.

In the analysis of methanolic extracts of *E. montanum* roots using HPLC-DAD, associated spectra were identified and quantified by means of reference standards of three phenolic acids, caffeic, chlorogenic and rosmarinic acids, with rosmarinic acid being a main component and exhibiting high levels of variation between cycles (Figure 1).

The concentrations of chlorogenic, caffeic and rosmarinic acids in *E. montanum* were significantly different (*p* < 0.01) between the sample origins (Po) and annual collection cycles (C). In addition, significant effects on the contents of chlorogenic and caffeic acids were observed for the interaction between locality of origin and annual cycle (Po × C). The linear models used in the data analysis revealed that for chlorogenic and caffeic acids, the magnitude of the effect of annual cycles, based on the variances, was greater than that of localities of origin and the Po × C interaction (Table 3). For rosmarinic acid, the locality of origin and annual cycle had a greater effect than the Po × C interaction, and the Po × C interaction effect was not significant, as observed for the chlorogenic and caffeic acid contents.

The response patterns of the concentrations of phenolic acids under natural growing conditions varied between annual cycles. For example, for chlorogenic acid, the prevailing environmental and edaphic conditions in 2021 favored a higher concentration than those in 2020, while the opposite pattern was observed for caffeic and rosmarinic acids (2020 > 2021). Varying effects of environmental conditions were also observed between localities, with micro-edaphoclimatic variations (Table 4) causing a higher concentration of phenolic acids in Morelos than in La Union. This result indicates that the site influenced the biosynthesis and accumulation of these compounds in the roots of *E. montanum*. The Po × C interaction did not have significant effects on the concentration of rosmarinic acid, indicating that the effect of locality was independent of that of the annual cycle (i.e., the change in concentration from one locality to another was independent of the changes in concentration from one annual cycle to another). The concentration of chlorogenic acid was higher in the 2021 cycle than in the 2020 cycle at both sampling locations. A higher concentration of caffeic acid was observed in Morelos in 2020, followed by La Union in 2020, indicating that in both locations, the edaphoclimatic conditions in 2020 supported a higher concentration than in 2021 (Table 4).

Comparing the effects of experimental levels or treatments within localities (Morelos and La Union) and annual cycles (2020 and 2021), it is pertinent to note that each study factor offered a contrast in ecogeographic, edaphic or response variability across locations or from quantification of environmental effects captured in the estimates of phytochemical composition, which we named as annual cycles. In the estimated effects as annual cycles, all climate elements and factors were synthesized without considering or evaluating independent effects, e.g., soil humidity, rainfall, soil fertility or altitude.

**Figure 2:**
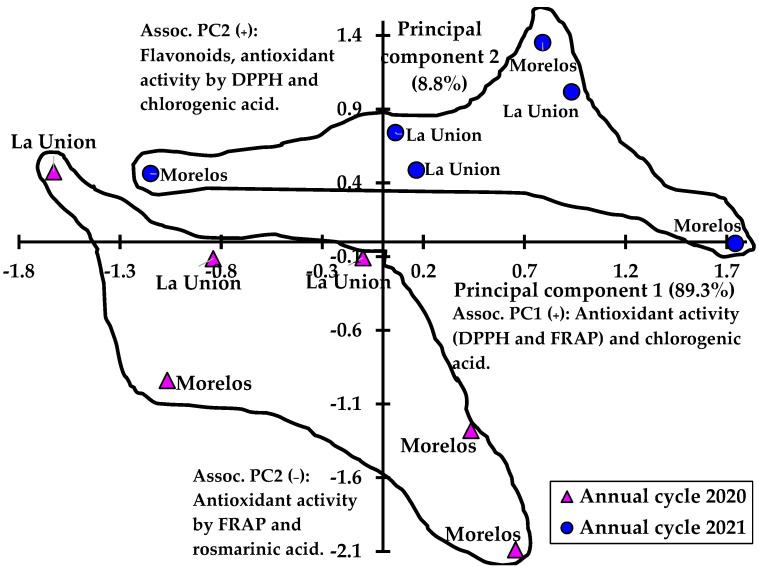
Scatterplot of collected samples of *E. montanum* roots from two communities and during two annual cycles (triangles 2020 and circles 2021), based on the first two main components of the analysis of phenolic compounds and antioxidant activity.

Based on the descriptive analysis of the main components, two distribution patterns associated with the annual cycles (2020 and 2021) were identified. Figure 2 shows the dispersion of samples by geographical origin and year of collection, where the samplings carried out in 2020 show a dispersion or distribution opposite to those carried out in 2021, and in general, these differences are associated with the contents of flavonoids and chlorogenic acid and antioxidant activity. The more pronounced multivariate differences between collection locations can be graphically distinguished more clearly in 2020 than in 2021. In addition, high variability is shown from one sampling year to another.

## 3. Discussion

For the results and effect analysis, a phenotypic model was considered, as proposed by Lynch and Walsh [22], where every compound evaluated (phenotypic value, P) is a result of genotypic, environmental and genotype–environment interaction effects (P = G + E + G × E). Much of the variation between locations (Po) was considered to result from genotypic effects, and the differences between annual growth cycles were attributed to overall environmental effects. The evaluation of methanolic extracts of *E. montanum* roots showed that the annual cycles (2020 and 2021) had a greater effect on the contents of total polyphenols and flavonoids and antioxidant activity than the localities of origin or sampling sites and the annual cycle–locality interaction (Table 1), indicating that the variations in the ecological–climatic conditions from one year to the next had a greater influence on the composition than the microniche and edaphic conditions at the localities. In this sense, Verma and Shukla [23] and Cheynier et al. [19] point out that the biosynthesis, transport and storage of phenolic compounds in plants is the product of the interaction of genetic, physiological and developmental or ontogenetic factors with environmental factors or growth conditions (e.g., drought, temperature, salinity or radiation stresses, seasonal variations and location) as part of the mechanisms of adaptive response and survival. In this work, the concentration of phenolic compounds in the samples collected in 2021 exceeded that in the samples collected in 2020, increasing from 13.2 to 33.9%, while the antioxidant activity in 2021 exceeded that in the samples collected in 2020, increasing from 8.8 to 18.7%, showing that the ecological conditions change from one year to another, as previously documented in the same Mixtec region by Rogé et al. [24], with increases in temperature and variations in seasonality and rainfall intensity. In this study, the results showed that phenolic compounds changed across annual cycles as part of the modifications in the metabolic biosynthesis and accumulation of this compound in the roots of *E. montanum*. In this sense, through a meta-analysis, Sun et al. [25] determined that increases in temperatures and decreases in annual rainfall increase the levels of phenolic compounds in medicinal and aromatic plants.

The site or locality of origin of the samples (Morelos or La Union) significantly influenced the contents of quercetin-equivalent flavonoids and antioxidant activity evaluated using the FRAP method. Higher flavonoid contents and antioxidant activity levels were recorded in Morelos than in La Union, and part of the effect can be attributed to differences in chemical composition of soil fertility. Higher concentrations of phosphorus, magnesium, and inorganic nitrogen but lower concentrations of sodium and iron were observed in the soils of Morelos than in the soils of La Union (Table 5). These results suggest that the fertility of soils or mineral constituents of the soil can influence the physiological and biochemical mechanisms of biosynthesis and accumulation of secondary metabolites in medicinal plants, as postulated by Hassan [26]. The findings follow a pattern similar to that described by Sampaio et al. [27] for *Lafoensia pacari* and Borges et al. [28] for *Myrcia tomentosa*, two medicinal species from Brazil; these authors emphasized that the ecological conditions and soil fertility in the sampling locations significantly influenced the contents of phenolic compounds.

The results showed independence of the effects of localities of origin and annual sampling cycles on the contents of flavonoids and antioxidant activity evaluated using DPPH. However, there were significant effects on the total polyphenol content and antioxidant activity evaluated using FRAP; for both parameters, the samples from 2021 had higher values than those from 2020 in both locations (Table 2). Based on these patterns, it is inferred that the contents of polyphenols, flavonoids and compounds associated with antioxidant activity in *E. montanum* roots were influenced by the effects of natural environmental conditions, which varied by locality and/or annual sampling cycle.

The microenvironmental or microniche conditions that occur in the sampling localities or distribution area of the species have a constant buffering or stress effect from one annual cycle to another that is not reflected in root composition. However, for the total polyphenols, the response was different, and the possible environmental stress under which the plants grew during the 2021 cycle caused an increase in the concentration in relation to that in the previous (2020) cycle. Genetic and genetic–environmental effects have not been ruled out, as suggested by Moore et al. [17] for secondary metabolites in general and Yang et al. [18], who point out that the response of plants to environmental factors through the synthesis and accumulation of metabolites cannot be attributed to a specific stress factor, either abiotic (e.g., solar radiation, temperature) or biotic (e.g., herbivores, competing plants), but to the interaction of all factors in a specific region that change from year to year. Based on the field observations, the sampled plants and roots were free of damages from pests or diseases.

Through HPLC-DAD analysis of the samples, rosmarinic acid, chlorogenic acid and caffeic acid were identified and quantified in the methanolic extracts of *E. montanum* roots. These compounds were also identified in extracts of *E. maritimum*, *E. campestre* and *E. planum*, and according to Cortés-Fernández et al. [5] and Kikowska et al. [8], they are associated with antioxidant properties that confer anti-inflammatory and antiprotozoal effects. Although these *Eryngium* species, including *E. montanum*, are used in traditional medicine for their anti-inflammatory, analgesic or antimicrobial properties, pharmacological and toxicity studies are lacking for most of them, especially regarding their root extracts [3,14]. However, for rosmarinic acid, identified as the main component of *E. montanum* extracts in this study, no toxicity in humans has been reported at doses of 100–200 mg/kg [14,29]. However, it is necessary to expand studies on this topic to guarantee the efficacy and safety of consuming these extracts.

In estimating the magnitude of the effects of the study factors through their variances or mean squares, it was determined that the effect of the annual sampling cycle (C) was greater than that of the locality of origin (Po) and that of the interaction between locality of origin and the annual cycle (Po × C): C > Po × C > Po for chlorogenic acid, C > Po > Po × C for caffeic acid and Po > C for rosmarinic acid (Table 4). The predominant effect of the annual cycle on the contents of phenolic acids suggests that annually, there are increases in temperature and variations in the rainy and dry seasons that induce these changes by modifying the edaphoclimatic characteristics of the sampling sites; however, these differences between years are not reflected in the annual climatological statistics, as documented by Rogé et al. [24] and Rogé and Astier [30].

Among the localities of origin, there were significant differences in the contents of chlorogenic, caffeic and rosmarinic acids, with the Morelos locality presenting higher values than La Union. Although these locations are less than 5 km apart, the microniche conditions and genetic and ontogenetic characteristics of the plants confer differences in phenolic acids in the roots of *E. montanum*. In addition to these intrinsic differences in the samples, there are also differences in the chemical composition of the sampled soils; Morelos is 100 m lower in altitude than La Union, but with higher contents of P, Mn, and N. In both places, the Ca content is high (39.3 to 40.5 cmol kg^−1^), which is related to low electrical conductivity (0.21 to 0.26 dS m^−1^) (Table 5); this soil condition generates absorption restrictions or stress on plants related to the absorption of nutrients, and therefore, the metabolic processes of phenolic acid biosynthesis are modified, accelerated or prevented, according to Banothu and Uma [31].

Between annual sampling cycles, significant differences in phenolic acid content were observed, with opposite tendencies. For example, the content of chlorogenic acid was 64.1% higher in the 2021 sample than in the 2020 sample; however, for caffeic acid and rosmarinic acid, the opposite pattern was observed, with 28.4% and 35.0% higher concentrations in 2020 than in 2021, respectively. These patterns suggest that multiple factors influence the synthesis and accumulation of phenolic acids. Yang et al. [18] noted that the concentration of metabolites in plants depends on the degree of environmental effect (e.g., light, temperature, water and soil fertility, salinity or alkalinity) on biochemical–physiological processes and their ability to synthesize secondary metabolites such as phenolic acids under conditions of seasonal stress. Bautista et al. [32] determined that the conditions (precipitation, salinity and soil physicochemical characteristics) in the growing season (spring, summer or autumn) of the Mediterranean region substantially modified the synthesis and accumulation of phenolic compounds in 19 wild species collected in five contrasting habitats. The results suggest that it is necessary to experimentally explore the individual effects of the factors that induce stress in plants and their effects on the biosynthesis and accumulation of phenolic acids; it is also expected that the response does not follow a linear cause–effect pattern for each compound.

The analysis of the locality–annual sampling cycle interaction (Po × C) revealed no significant effect of this interaction on the concentration of rosmarinic acid, indicating that the effects were independent. In contrast, for chlorogenic and caffeic acids, the effect of the interaction was significant; that is, they were affected by the locality and the annual environmental conditions (Table 4). The variation in the concentration of rosmarinic acid due to the Po × C interaction ranged from 5011.3 to 9674.8 µg g^−1^. Hatami et al. [33] also identified a large amount of variation in the contents of rosmarinic acid in *Eryngium caucasicum* Trautv in six geographic regions of Iran (ranging from 0.118 to 1.234 µg g^−1^) by means of HPLC, and their results showed high levels of variation within each geographic location of origin, with a coefficient of variation equal to 50.81%. In contrast, Kikowska et al. [34] estimated 495 and 4309 µg g^−1^ concentrations of rosmarinic acid in the roots of *E. maritimun* L. grown in the field and in seedlings grown in vitro, respectively. All these estimates indicate that the synthesis, translocation and accumulation of rosmarinic acid in species of the genus *Eryngium* are a product, in part, of the conditions in which the individual plants grow and the organs from which the analyzed samples are obtained.

The concentration of chlorogenic acid in the methanolic extracts of *E. montanum* roots increased from 2020 to 2021, with the same pattern at both sampling sites (Morelos and La Union), indicating that the effects of season or annual edaphoclimatic conditions could be greater, depending on the locality or geographical area, even though in this case, the collection sites were less than 10 km apart. For caffeic acid, the same trend was observed between the sampling locations, but the concentration pattern was reversed (higher in 2020 than in 2021). Such changes in the concentration of chlorogenic and caffeic acids were also observed by Kikowska et al. [35] in *E. planum* L. For example, higher concentrations of chlorogenic acid were recorded in roots of seedlings grown in the field than in roots of seedlings grown in vitro; the pattern for caffeic acid was the opposite, with a lower concentration in plants grown in the field and a higher concentration under in vitro conditions. These responses suggest that environmental conditions determine the concentrations of phenolic acids, but the changes in compounds are independent.

The contrasting patterns for the concentrations of chlorogenic and caffeic acids suggest that the metabolic responses of the roots are a product of the prevailing conditions during the recent rainy season or previous annual cycle, since the roots remain in the soil and the plants do not produce shoots or stems until favorable humidity conditions occur, which are part of the plant’s survival strategy during prolonged periods of drought and limited humidity [36,37]. Therefore, the biosynthesis and accumulation of phenolic acids (chlorogenic, caffeic and rosmarinic acids) in the roots of *E. montanum* were sensitive to edaphic and climatic conditions at each site and during the annual growth cycles. However, it is necessary to determine the individual environmental factors affecting the biosynthesis and accumulation of specific phenolic compounds in *E. montanum*, such as soil fertility, humidity and temperature, as well as ontogenetic factors such as plant age, phenological stage and harvest season. Additionally, toxicity and pharmacological evaluations of *E. montanum* root extracts should be performed in future studies.

In response to climate change, plants are continuously exposed to stress conditions (e.g., extreme temperature, drought, salinity). Then, reactive oxygen species (ROS) mechanisms and enzymatic systems act to maintain homeostasis within the cells and different defense systems are activated in the cells and organs of the plant, such as the synthesis of antioxidant compounds or redox reactions [38,39], including those related to all phenolic compounds evaluated in this study. For future studies, we suggest evaluating the direct relationships between specific stressors (e.g., drought or extreme temperature) and antioxidant enzyme activity or specific phenolic compounds under controlled experimental conditions. In addition, to date, there have been no recommendations for the use of *E. montanum* for human consumption (e.g., USA-FDA, FAO/WHO or other organizations) because it is in the experimental phase.

## 4. Materials and Methods

### 4.1. Plant Material

**Figure 3:**
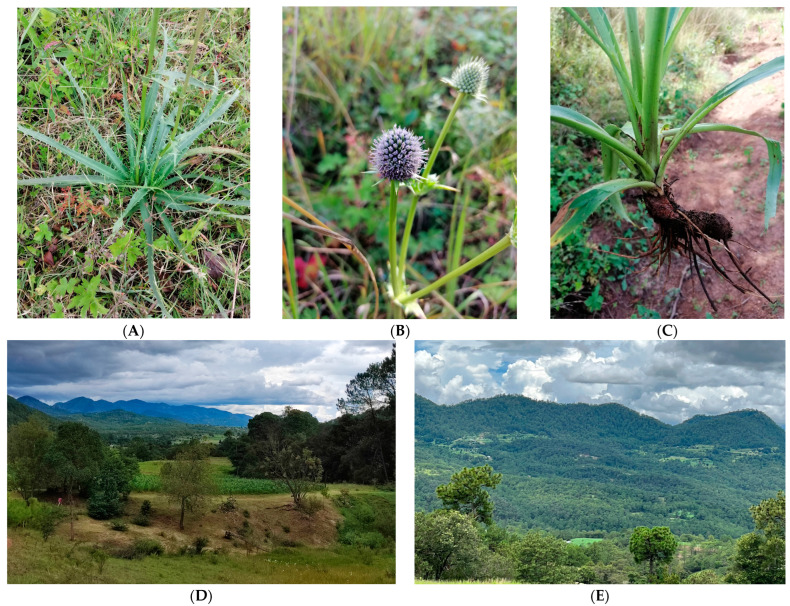
*Eryngium montanum* JM Coult. & Rose growing under natural conditions in San Martin Huamelulpam, Oaxaca, Mexico: (**A**) plant, (**B**) inflorescence, (**C**) root. (**D**) Morelos location and (**E**) La Union location (photos by M.L.P.O).

*Eryngium montanum* JM Coult. & Rose is a perennial plant endemic to central and south–southeastern Mexico, and to date, *E. montanum* has not been included on the Red List of the International Union for Conservation of Nature (IUCN) [15]. The plants have 1 m high floral axes, inflorescences composed of capitula with embedded individual purple flowers, bracts and basal leaves in the form of a rosette with spiny edges and thinner secondary branches; the taproots, sometimes branched or fasciculate, are formed by a group of secondary roots that have the same or similar thicknesses [40] (Figure 3). The taxonomic identification of the species was confirmed by a taxonomist of the Instituto de Biologia of the Universidad Nacional Autonoma de Mexico. This species is distributed on the Pacific coast from Oaxaca to Nayarit, Mexico [11], and it is common to find it in the high temperate zones on the margins of or within *Pinus* and *Quercus* forests, tributaries and farmland with relatively high humidity during the growth phase (e.g., with stems, leaves and inflorescences) [40]. In San Martin Huamelulpam, Oaxaca, it is known as ‘espina de burro’ or ‘maguey de burro’ (Spanish), and the roots are used in infusions for the treatment of diarrhea, stomach pain and various other gastrointestinal disorders.

### 4.2. Experimental Design and Sampling of Plants for Analysis

*E. montanum* root samples for phytochemical analysis were obtained from plants in the flowering stage after the rainy season. Sampling of *E. montanum* roots for phytochemical analysis was carried out in Morelos and La Union, two sites or localities within the municipality of San Martin Huamelulpam, Oaxaca, where the plant populations were abundant (Figure 3). In the sampling locations, *E. montanum* plants grow in disturbed places such as forest clearings and near crop fields in the margins of or within pine–oak forests. The associated plant communities include low-growing shrubs and herbaceous plants, generally from the *Poaceae* family and other *Eryngium* species, and species of the *Ageratina*, *Geranium*, *Salvia* and *Tagetes* genera (Figure 3). *E. montanum* root samples were obtained from plants in the flowering stage after the rainy season. According to our field observations, the samples of *E. montanum* were free of visible damage from insects and fungal diseases. At the location level, the whole roots of eight to ten individual plants were collected in three population patches of *E. montanum* in Morelos and three in La Union (three compound samples/location) in October 2020. The second year, we returned to the same patches and, again, three compound samples per location were taken (October 2021). Later, each compound sample was dried and ground for spectrophotometric and HLPC analysis. As the same population patches were sampled during two annual cycles, we ensured the same populations or subpopulations were sampled, but without evidence to assume genetic homogeneity among and within the sampled populations. A bifactorial arrangement of treatments was applied. In the first level (Factor A), sites were grouped according to the ecogeographic origin and natural growing conditions of the populations sampled; sites were grouped into Morelos and La Union within the municipal territory of SM Huamelulpam (Po, populations). The second factor (Factor B) was the annual growth cycle (C), with two levels: 2020 and 2021. Consequently, we assumed a two-factor design, where Factor A represented the locations where the plants were obtained in situ and Factor B represented the growth conditions in each annual cycle, considering a completely randomized experimental design. Additionally, soil samples were taken at both sampling locations for chemical analysis. Descriptions of the collection sites, climatic conditions and soil characteristics are provided in Table 5.

**Table 5:** Geographic and environmental growing conditions of *E. montanum* sampled in Oaxaca, Mexico.

Geographic and Environmental Growing Conditions of *E. montanum*	San Martin Huamelulpam, Oaxaca, Mexico	
	Morelos	La Union
Locality descriptors:		
Elevation (m)	2200	2301
Latitude (N)	17°23′43.9″	17°23′16.3″
Longitude (W)	97°35′16.1″	97°37′09.8″
Annual average temperature (°C) ^a^	14–18	
Annual average rainfall (mm)	700–1000	
Solar radiation (W/m^2^, maximum)	1155–1308	
Climate (predominant)	Temperate subhumid with precipitation from June to November	
Soil chemical analyses ^b^		
Organic matter (%)	1.81	1.83
pH (in H_2_O)	8.00	8.24
P-Olsen (mg kg^−1^)	4.37	1.29
B-Olsen (mg kg^−1^)	0.40	0.41
K (cmol kg^−1^)	0.66	0.76
Ca (cmol kg^−1^)	40.5	39.3
Mg (cmol kg^−1^)	1.20	1.35
Na (cmol kg^−1^)	0.08	0.11
Fe (mg kg^−1^)	3.61	5.20
Zn (mg kg^−1^)	0.34	0.22
Mn (mg kg^−1^)	18.66	7.38
Cu (mg kg^−1^)	0.61	0.67
Inorganic N (mg kg^−1^)	7.76	1.90
Electrical conductivity (dS m^−1^)	0.26	0.21
Cation exchange capacity (CEC) (cmol kg^−1^)	43.0	42.0

^a^ INEGI [41]; ^b^ soil analyses performed according to current Mexican standards (NOM-021-RECNAT-2000) [42].

### 4.3. Evaluation of Phenolic Compounds and Antioxidant Activity Using Spectrophotometry

#### 4.3.1. Sample Preparation

*E. montanum* roots were rinsed, cut and dried at 40 °C in a dehydrator (L’Equipe Model 528). The samples were then pulverized using an electric grinder (Moongiantgo^®^, model HO-150, WA, USA) and stored at −20 °C in an airtight amber container until evaluation. Methanolic extract was obtained from 0.1 g of dry ground sample and 25 mL of 60% (*v*/*v*) methanol, which was homogenized for 60 s at 30 s intervals (Ultra Turrax T 25 Digital, IKA, Staufen, Germany) and centrifuged at 11,000 rpm (Eppendorf AG, Mod. 5811F, Hamburg, Germany) and 4 °C for 15 min. The supernatant was used for the spectrophotometric determinations of phenolic compounds and antioxidant activity (two methanolic extracts), and all determinations were carried out in triplicate.

#### 4.3.2. Total Polyphenol Content

The total polyphenol content was estimated according to the Folin–Ciocalteu method described by Singleton and Rossi [43], with some modifications as detailed in a previous study [44]. The results are expressed as milligrams of gallic acid equivalent per gram of dry weight (mg GAE g^−1^ dw).

#### 4.3.3. Flavonoid Content

The determination of the flavonoid content was carried out using two colorimetric methods following the methodologies proposed by Lin and Tang [45] and Zhishen et al. [46] with modifications described in Pérez-Ochoa et al. [44]. The results were expressed per gram of dry weight as quercetin equivalent milligrams (mg QE g^−1^ dw) and catechin equivalent milligrams (mg CE g^−1^ dw).

#### 4.3.4. Antioxidant Activity

Two methods were used to determine the antioxidant activity of the extracts. The first was the radical scavenging of DPPH described by Brand-Williams et al. [47], and the second was the reducing power of iron through the FRAP method described by Benzie and Strain [48] with modifications presented in a previous study [44]. The results of both determinations were expressed as micromoles of Trolox equivalents per gram of dry weight (μmol TE g^−1^ dw).

### 4.4. Evaluation of Phenolic Compounds Using HPLC-DAD

Sample preparation. Methanolic extracts were obtained from 0.3 g of dry and ground root samples in 15 mL of 60% (*v*/*v*) methanol, homogenized twice for 30 s (Ultra Turrax T 25 Digital, IKA, Staufen, Germany) and sonicated in an ultrasonic bath (Cole-Parmer, Mod. 08895-43, Vernon Hills, IL, USA) for one hour. Subsequently, the sample was centrifuged at 11,000 rpm (refrigerated Eppendorf 5810R centrifuge) for 15 min at 4 °C, and the supernatants were recovered. For HPLC-DAD analysis, a 2 mL aliquot of methanolic extract (60%) was filtered using a 0.2-μm PTFE syringe (Agilent Technologies^®^, Part. No. 5190-5086, Waldbronn, Germany) and placed in amber glass vials before injection. For the analysis, we used two methanolic extracts per compound sample with double injection or reading.

HPLC-DAD analysis. The analysis of phenolic acids and flavonoids was performed using an HPLC instrument (Agilent model Infinity II 1260 LC system) equipped with a solvent degasser, quaternary pump, temperature-controlled autosampler, column oven and DAD (Agilent Technologies, Santa Clara, CA, USA) and a reversed-phase column (Agilent^®^ Hypersil 5 ODS, 250 × 4.6 mm, 5 μm) following the method described by Pająk et al. [49] with some modifications as described in Pérez-Ochoa et al. [44]. The monitoring wavelength for caffeic, chlorogenic and rosmarinic acids was 320 nm. Each compound was identified with reference to the retention times and UV spectra of commercial standards (Sigma-Aldrich^®^, St. Louis, MO, USA) and quantified using the following calibration curves of reference standards: caffeic acid (0.04 to 12 µg mL^−1^, r^2^ = 0.999), chlorogenic acid (1.27 to 203 µg mL^−1^, r^2^ = 0.998) and rosmarinic acid (0.9 to 533 µg mL^−1^, r^2^ = 0.999). The amount of each compound was expressed as micrograms per gram of dry weight (µg g^−1^ dw).

### 4.5. Statistical Analysis

Analysis of variance based on a completely random linear model was carried out with the information obtained from the phytochemical analysis of the samples [22]. Comparisons of means were made by Tukey’s test (*p* < 0.05) to evaluate the specific differences between sample origins (Po, populations) and annual cycles (C, 2020 and 2021) and due to locality–annual cycle interactions (Po × C). Additionally, Pearson correlation analysis of total polyphenols and flavonoids with antioxidant activity according to the annual cycles was carried out, and principal component analysis was performed using the average values per sample. All statistical analyses were performed with the SAS statistical package [50].

## 5. Conclusions

Spectrophotometric analysis showed that the contents of polyphenols and flavonoids and antioxidant activity in roots of *Eryngium montanum* were significantly influenced by the environmental conditions during the annual growth cycles, the localities of origin of the samples influenced the contents of quercetin-equivalent flavonoids and antioxidant activity evaluated using FRAP and the interaction between the annual growth cycle and locality of origin was significant only for the total polyphenols and antioxidant activity evaluated by FRAP. Analysis of *E. montanum* root extracts through HPLC-DAD was used to identify and quantify chlorogenic acid, caffeic acid and rosmarinic acid. The concentrations of these metabolites were influenced by the locality of origin, annual growth cycle and the interaction between locality and annual cycle, except for rosmarinic acid, for which the interaction was not significant. In all cases, the effects of the annual growth conditions on the concentrations of phenolic acids were greater than the effects of locality and the interaction between locality and annual cycle; that is, the annual edaphoclimatic conditions significantly influenced the synthesis, translocation and accumulation of phenolic acids in the roots of *E. montanum*. The set of bioactive compounds contained in the roots of *E. montanum* partially explains the therapeutic properties of the roots in the treatment of gastrointestinal disorders among native communities of Oaxaca, Mexico.

## Figures and Tables

**Table 1:** Significance of the mean squares from the analysis of variance of phenolic compounds and antioxidant activity in *E. montanum* roots from Oaxaca, Mexico.

Sources of Variation	Total Polyphenols	Flavonoids		Antioxidant Activity	
		QE ^3^	CE ^3^	DPPH	FRAP
Annual cycle (C) ^1^	620.1 **	24.9 **	1634.0 **	25,738.0 **	16,833.1 **
Localities of origin (Po)	3.3 ^ns^	5.8 **	12.0 ^ns^	178.3 ^ns^	13,852.8 **
Po × C	80.4 **	1.7 ^ns^	19.8 ^ns^	17.1 ^ns^	13,124.7 **
Sampling (S)	365.9 **	7.6 **	219.5 **	10,032.6 **	30,027.8 **
Lab replicates/S ^2^	5.4 ^ns^	0.3 ^ns^	2.0 ^ns^	96.3 ^ns^	588.2 ^ns^
Error	8.6	0.5	6.9	248.2	907.6
Coeff. variation (%)	6.1	12.9	8.0	7.1	8.3

^1^ Annual cycle (2020 and 2021). ^2^ Nesting of laboratory replicates within samplings; ^3^ QE and CE indicate quercetin and catechin equivalents, respectively; ^ns^ not significant (*p* > 0.05); ** significant at *p* < 0.01.

**Table 2:** Effects of the annual cycles and localities of origin on the phenolic compound contents and antioxidant activity of *E. montanum* roots collected in Oaxaca, Mexico.

Study Factors	Levels	Total Polyphenols (mg GAE g^−1^ dw)	Flavonoids (mg g^−1^ dw)		Antiox. Activity (µmol TE g^−1^ dw)	
			QE ^1^	CE ^1^	DPPH	FRAP
Annual cycle (C)	2020	44.7 ^b 2^	4.9 ^b^	28.2 ^b^	202.3 ^b^	349.2 ^b^
	2021	50.6 ^a^	6.1 ^a^	37.7 ^a^	240.1 ^a^	379.8 ^a^
Localities of origin (Po)	Morelos	47.4 ^a 2^	5.8 ^a^	33.3 ^a^	222.8 ^a^	378.4 ^a^
	La Union	47.8 ^a^	5.2 ^b^	32.5 ^a^	219.7 ^a^	350.6 ^b^
Locality–annual cycle (Po × C) interactions:						
Morelos	2020	45.5 ^b 2^	5.0 ^a^	28.0 ^a^	203.4 ^a^	376.6 ^a^
	2021	49.3 ^a^	6.5 ^a^	38.6 ^a^	242.2 ^a^	380.2 ^a^
La Union	2020	43.9 ^b^	4.8 ^a^	28.3 ^a^	201.2 ^a^	321.8 ^b^
	2021	51.8 ^a^	5.6 ^a^	36.8 ^a^	238.1 ^a^	379.4 ^a^

^1^ QE and CE indicate quercetin and catechin equivalents, respectively. ^2^ In columns, means for annual growth conditions, localities or interactions between annual cycle and locality followed by the same letter are not significantly different (Tukey’s test, *p* < 0.05).

**Table 3:** Significance of mean squares from the analysis of variance of phenolic acids identified in *E. montanum* roots using HPLC-DAD.

Source of Variation	Chlorogenic Acid	Caffeic Acid	Rosmarinic Acid
Annual cycle (C)	60,039,514 **	2638.8 **	57,150,509 **
Localities of origin (Po)	90,211 **	829.2 **	73,876,241 **
Po × C ^1^	3,877,909 **	170.6 **	21,042 ^ns^
Sampling (S)	10,828,390 **	210.9 **	20,703,632 ^ns^
Rep./S ^2^	8440 ^ns^	20.4 *	1,417,430 ^ns^
Error	4929	4.7	1,113,390
Coeff. variation (%)	1.5	3.6	14.5

^1^ Interactions between locality of origin and annual cycle. ^2^ indicates nesting of laboratory replicates within samplings. * significant at *p* < 0.05; ** significant at *p* < 0.01; ^ns^ not significant (*p* > 0.05).

**Table 4:** Phenolic acid contents identified using HPLC-DAD in *E. montanum* roots collected under natural conditions in two consecutive years.

Compounds Evaluated (µg g^−1^ dw)	Annual Cycle (C)		Localities of Origin (Po)		Locality–Annual Cycle Interactions (Po × C)			
					Morelos		La Union	
	2020	2021	Morelos	La Union	2020	2021	2020	2021
Chlorogenic acid	3488.5 ^b 1^	5725.3 ^a^	4650.2 ^a^	4563.5 ^b^	3247.6 ^b^	6052.9 ^a^	3729.3 ^b^	5397.7 ^a^
Caffeic acid	67.4 ^a^	52.5 ^b^	64.1 ^a^	55.8 ^b^	69.6 ^a^	58.6 ^c^	65.1 ^b^	46.5 ^d^
Rosmarinic acid	8413.3 ^a^	6230.9 ^b^	8562.7 ^a^	6081.5 ^b^	9674.8 ^a^	7450.6 ^a^	7151.7 ^a^	5011.3 ^a^

^1^ Within rows, values with the same letter did not differ significantly between localities of origin, between annual growth conditions or due to the interaction between locality and annual growth conditions (Tukey’s test, *p* < 0.05).

## Data Availability

Not applicable. Data are contained within the article.
